# Frontoparietal Structural Connectivity Mediates the Top-Down Control of Neuronal Synchronization Associated with Selective Attention

**DOI:** 10.1371/journal.pbio.1002272

**Published:** 2015-10-06

**Authors:** Tom Rhys Marshall, Til Ole Bergmann, Ole Jensen

**Affiliations:** 1 Donders Institute for Brain, Cognition and Behaviour, Radboud University Nijmegen, Nijmegen, The Netherlands; 2 Institute of Psychology, Christian-Albrechts University of Kiel, Kiel, Germany; University of Oxford, UNITED KINGDOM

## Abstract

Neuronal synchronization reflected by oscillatory brain activity has been strongly implicated in the mechanisms supporting selective gating. We here aimed at identifying the anatomical pathways in humans supporting the top-down control of neuronal synchronization. We first collected diffusion imaging data using magnetic resonance imaging to identify the medial branch of the superior longitudinal fasciculus (SLF), a white-matter tract connecting frontal control areas to parietal regions. We then quantified the modulations in oscillatory activity using magnetoencephalography in the same subjects performing a spatial attention task. We found that subjects with a stronger SLF volume in the right compared to the left hemisphere (or vice versa) also were the subjects who had a better ability to modulate right compared to left hemisphere alpha and gamma band synchronization, with the latter also predicting biases in reaction time. Our findings implicate the medial branch of the SLF in mediating top-down control of neuronal synchronization in sensory regions that support selective attention.

## Abstract 2

Fiber tracts that connect regions in the front and back of the brain provide control signals to direct the oscillatory activity associated with paying attention to visual stimuli.

## Author Summary

Directing attention to a part of visual space produces patterns of "brainwaves" or neuronal oscillations in the human visual cortex (the part of the brain at the back that processes incoming information from the eyes); oscillations at low frequencies are believed to help the brain block out irrelevant or distracting information, whereas high-frequency oscillations signal processing of relevant information. The instructions to increase or decrease these oscillations likely originate in the front part of the brain. In this study, we investigated the structural "highways"—bundles of white matter—that connect the front and back of the brain together. Not only did we show that these highways are asymmetric—i.e., some participants have a larger fiber bundle in the left hemisphere of their brains, and some in the right—we also showed that these asymmetries predicted whether subjects were better able to control the neuronal oscillations in their left or right hemispheres. This, in turn, predicted whether the participants were faster in detecting targets in the right or left half of the screen. Thus, we showed that these structural highways are important in helping the brain pay attention to parts of visual space.

## Introduction

In order to operate in complex environments, it is necessary to selectively attend to relevant information while inhibiting distraction. It has been proposed that changes in neuronal synchronization implement the mechanism required for selective gating [1,2]. The increase in synchronization supports a gain increase [3] as well as information transfers to downstream regions by means of communication through coherence [4]. For instance, neurons in the monkey visual cortex activated by a given object show increased gamma-band (50–90 Hz) synchronization when attention is allocated to that object [1,5]. These results generalize to human electroencephalography (EEG) and magnetoencephalograhy (MEG) studies that have identified increased gamma band activity associated with selective attention [6–8]. Alpha oscillations on the other hand have been proposed to reflect active inhibition of distracting information. This is underscored by alpha oscillations (8–12 Hz) being relatively strong in regions anticipating distracting input [9–11]. Modulations in both the alpha and gamma band are predictive of performance in visual attention tasks [6,12–14]. Given that these neuronal oscillations are modulated by selective attention, they are under top-down control. The aim of this study is to identify the anatomical pathways supporting the top-down control of the oscillatory activity in sensory regions.

Cue-directed shifts of attention are believed to be subserved by the dorsal attentional network [15] consisting of the frontal eye field (FEF) and intraparietal sulcus (IPS), in contrast to the ventral attentional network that governs stimulus-driven attentional shifts [15]. Recent studies using transcranial magnetic stimulation (TMS) have implicated the dorsal network in providing top-down control of alpha [16–18] and gamma [18] oscillations. Communication within the dorsal network must be subserved by structural connections, and there is evidence that the development of frontoparietal white matter tracts co-occurs with recruitment of superior frontal and parietal cortex during attention and working memory tasks [19,20]. The superior longitudinal fasciculus (SLF), a network of white-matter fiber tracts consisting of medial, middle, and lateral branches [21], has recently been proposed to connect prefrontal control areas to posterior regions. In particular, the medial SLF branch (SLF1) projects to areas overlapping with the dorsal network—namely posterior superior frontal cortex in and near to the FEF and the IPS [21]. The lateral branch (SLF3) projects to nodes in the ventral network (inferior frontal gyrus and temporoparietal junction [21]), while the middle branch (SLF2) supposedly provides connections between the two networks. Individual differences in SLF2 volume have been shown to predict behavioral attentional biases [21,22]. Further, the number of SLF1 connections predicts the disruptive effects of FEF perturbation with TMS on visual task performance [23]. Given that individual differences in the SLF are behaviorally relevant, we hypothesize that the variance in these tracts also explains individual abilities to modulate alpha and gamma oscillations in sensory regions.

In the present study, we performed both MEG and high angular resolution diffusion imaging (HARDI) magnetic resonance (MR) measurements in the same subjects. Oscillatory brain activity was quantified from the MEG data while the subjects performed a cued spatial attention task requiring attention to the left or right visual hemifield. From the MR data, we used whole-brain spherical deconvolution tractography [24,25] to reconstruct the SLF branches. We hypothesized that the medial branch (SLF1)—connecting superior frontal to parietal cortex [21]—served as the structural pathway for controlling oscillatory brain activity in visual brain regions. Therefore, individual differences in SLF1 properties should predict individual ability to modulate visual cortical oscillations and thereby performance on a spatial attention task.

## Results

**Fig 1:**
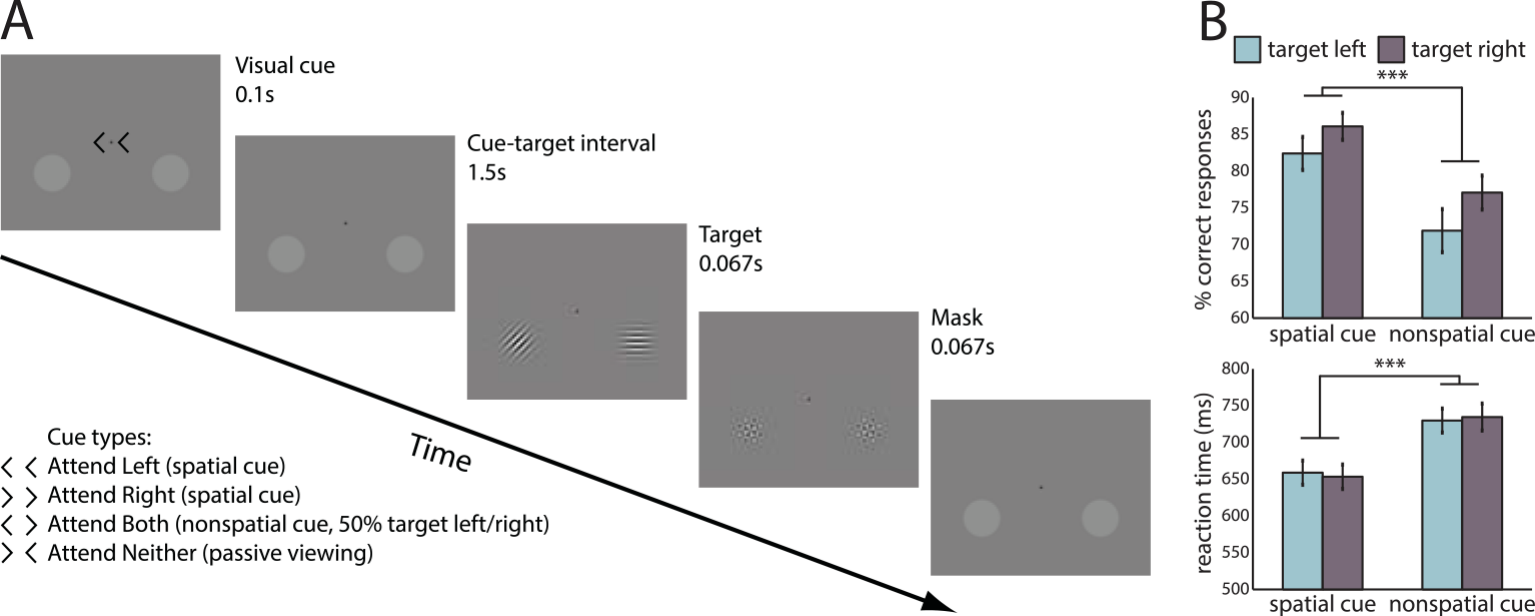
(A) Experimental paradigm. Each trial began with one of four visual cues, instructing the subject either to attend to the left luminance pedestal, the right luminance pedestal, to both luminance pedestals, or to passively fixate. After a 1.5 s fixed interval, a pair of Gabor patches appeared in both luminance pedestals. One Gabor patch was always diagonally oriented (45° clockwise or counterclockwise from vertical), and the other cardinally oriented (horizontal or vertical). In the "attend left" and "attend right" conditions, the diagonal patch appeared respectively in the left or right pedestal; in the "attend both" and "attend neither" conditions, location of the diagonal patch was random. Subjects had to discriminate the orientation of the diagonal patch. (B) Analysis of behavioral data revealed that spatial cueing significantly improved both reaction time and accuracy, whereas target hemifield did not alter reaction time or accuracy.

We acquired data from 26 subjects. These subjects performed a cued attention task in the MEG requiring shifts of attention to the left, right, or to both visual hemifields in order to identify the orientation of an upcoming target grating briefly presented 1,500 ms after the cue (Fig 1A). A second grating was always concurrently presented in the unattended hemifield. Analysis of the behavioral data using repeated-measures ANOVA confirmed that spatial cueing improved both accuracy and reaction time, respectively by 10% and 76 ms (Fig 1B; accuracy: F(1,25) = 42.077, *p* < 10^−6^; reaction time: F(1,25) = 110.114, *p* < 10^−9^). Direction of attention did not significantly alter these variables, and no interaction of direction with cueing was observed (*p* > 0.05 in all cases).

### Anticipatory Alpha and Stimulus-Induced Gamma Demonstrate Attentional Modulation

**Fig 2:**
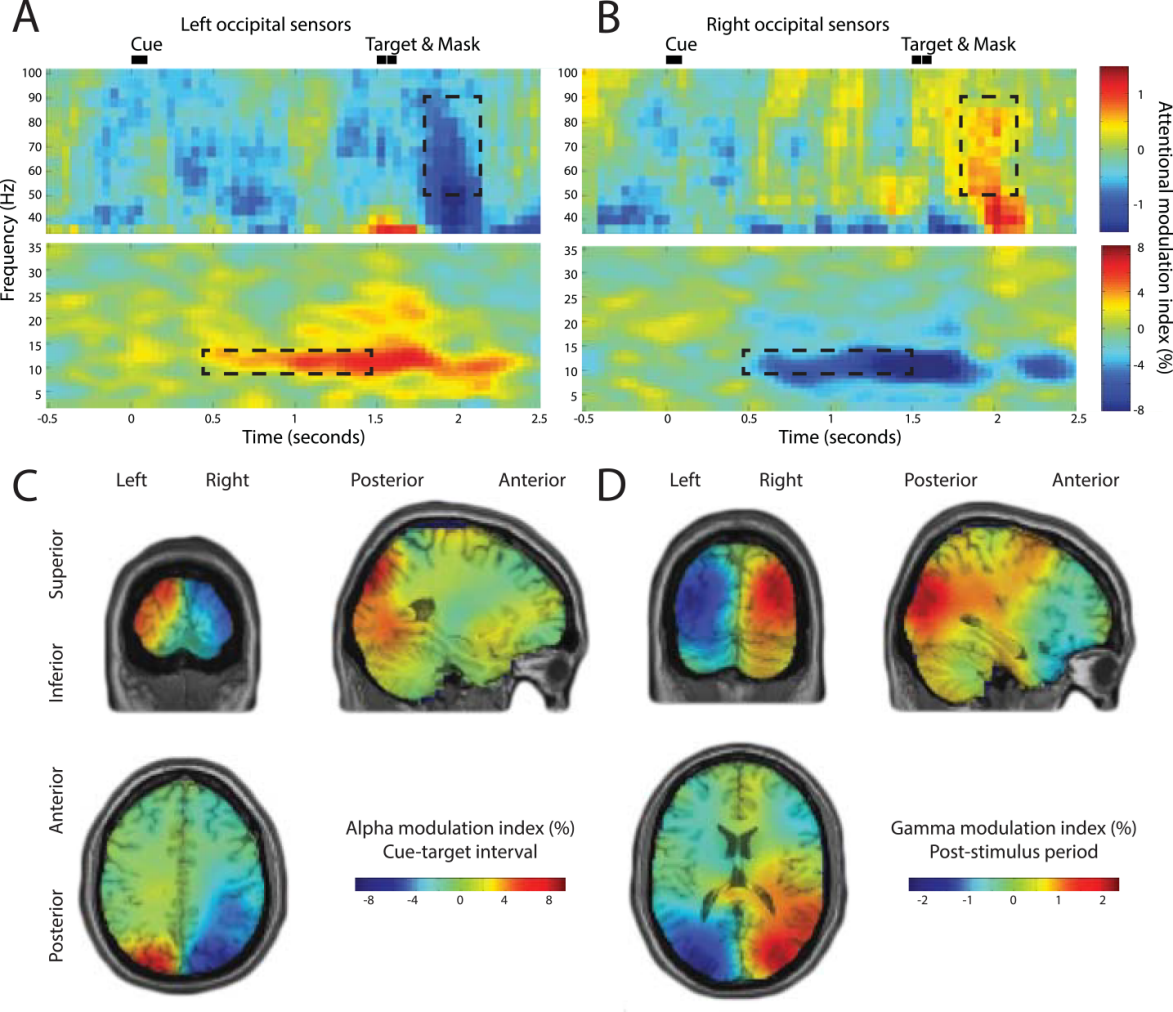
Time-frequency analysis and source reconstructions of attentional modulation of anticipatory alpha and stimulus-induced gamma oscillations. (A,B) For left and right occipital MEG sensors. “Attention left” trials were compared to “attention right” trials. Bilateral attentional modulation is clearly visible in the alpha band during the cue-target interval, and bilateral modulation of stimulus-induced gamma oscillations is clearly visible during the post-stimulus interval. (C) Grand average alpha modulation index (attention left versus attention right) calculated for cue-target interval (350–1,350 ms post-cue); alpha modulation is strongest in the bilateral superior occipital cortex. (D) Grand average gamma modulation index calculated for post-stimulus interval (1,700–2,100 ms post-cue); gamma modulation is strongest in the bilateral middle occipital cortex.

We first confirmed previous results demonstrating that both anticipatory alpha oscillations (defined as 8–12 Hz activity in a 1 s window prior to presentation of the target and distractor stimuli) and stimulus-induced gamma activity (defined as 50–90 Hz activity in a 400 ms window following target and distractor presentation) in occipital brain regions are modulated by direction of attention. Attentional modulation index (AMI) was calculated for each sensor *j* according to the formula *AMI*_*j*_ *=* 100% * *(Power*_*Attention left*,*j*_ *—Power*_*Attention right*,*j*_ *) / (Power*_*Attention left*,*j*_ *+ Power*_*Attention right*,*j*_ *)*. The sensor-level analysis revealed a robust increase in gamma band activity in response to the target contralateral to the attended hemifield (Fig 2A and 2B). This finding is consistent with gamma band synchronization reflecting visual processing that is modulated by selective attention. The alpha band activity was strongly modulated in the cue-target interval and showed a relative decrease contralateral to the attended hemifield. The strong modulation during this delay is consistent with the notion that alpha band activity reflects the anticipatory allocation of attentional resources. No strong attentional modulation was observed in the intermediate beta-band or in other frequency bands.

To determine the underlying cortical sources of these modulations, we used a frequency domain spatial filtering technique (a beamformer approach [26]). To statistically quantify these modulations we used cluster-based permutation statistics [27], a method controlling for multiple comparison in space (see Materials and Methods). When comparing power values from “attention left” and “attention right” trials, we found robust modulations in the occipital cortex. When subjects were cued to the left, right occipital alpha power was lower than when they were cued to the right. The reverse pattern was observed in the left hemisphere (Fig 2C). These differences were greatest in the superior occipital cortex (MNI coordinates: left, −26 −92 38, right, 34 −82 44; associated clusters: left, *p* = 0.02; right, *p* = 0.0008, see S1 Fig). Conversely, when subjects were cued to the left, right occipital gamma power was higher than when they were cued to the right, and the reverse pattern was observed in the left hemisphere (Fig 2D). These differences were greatest in the middle occipital cortex (MNI coordinates: left hemisphere, −26 −94 16; right hemisphere, 34 −82 16; associated clusters: left, *p* = 0.002; right, *p* = 0.004, see S2 Fig). Consistent with the literature, both anticipatory alpha oscillations and stimulus-induced gamma band activity in occipital cortex are robustly modulated by spatial attention [6–12].

### Hemispheric Asymmetry of the SLF1 Correlates with Hemispheric Asymmetry of Both Alpha and Gamma Band Activity

**Fig 3:**
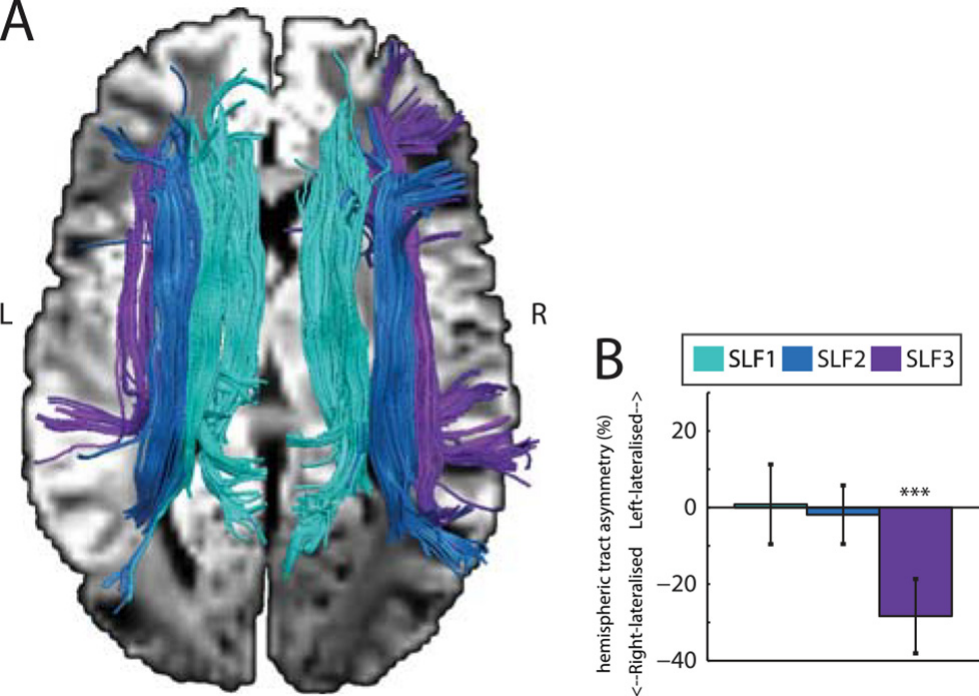
(A) Tractographic rendering of SLF branches in one subject obtained using diffusion MRI. The medial branch (SLF1) is shown in sky blue, the middle branch (SLF2) is shown in dark blue, and the lateral branch (SLF3) is shown in purple. These branches were identified by following the tracts intersecting coronal slices passing through both parietal cortex and, respectively, the superior frontal gyrus (SLF1), middle frontal gyrus (SLF2), and precentral gyrus (SLF3). (B) Group average hemispheric tract asymmetry for the three SLF branches. Consistent with previous work [21], only SLF3 shows consistent right lateralization (t(25) = -6.02, *p* < 0.0001). SLF1 and SLF2 are not lateralized (SLF1: t(25) = 0.17, *p* = 0.87. SLF2: t(25) = -0.51, *p* = 0.62). Error bars represent 95% confidence intervals. *** indicates *p* < 0.0001.

Next, we sought to relate individual differences in modulations of the gamma and alpha band activity to properties of the SLF. Spherical deconvolution tractography [25,28] was used to reconstruct the SLF branches from the diffusion data. Consistent with previous research [21,23], a network of three branches in each hemisphere was reconstructed (Fig 3A). For each of the three SLF branches, a hemispheric asymmetry index was computed (100% *(volume_left–volume_right)/ (volume_left + volume_right);* see Materials and Methods), quantifying whether each subject had greater tract volume in the left or right hemisphere. Nonoverlapping regions were identified as regions of interest (ROIs) in prefrontal cortex and then used for seeding the fiber tracking. This ensured that the fiber bundles were well separated. The medial SLF1 branches were defined as fibers passing through superior frontal gyrus, SLF2 as passing through middle frontal gyrus, and SLF3 as passing through precentral gyrus (see Materials and Methods). Replicating previous findings [21], the SLF3 was right-lateralized at the group level, whereas SLF1 and SLF2 did not show evidence of lateralization at the group level (see Fig 3B). Furthermore, a modulation asymmetry index was also calculated for each subject’s MEG data indicating whether—for both alpha and gamma oscillations—that subject displayed a stronger degree of power modulation with attention in the left or right hemisphere (*ΔAMI = (- AMI*_*left*,*j*_ *)—AMI*_*right*,*j*_; see Materials and Methods). We derived the alpha and gamma modulation values (*ΔAMI*) from the anatomical regions demonstrating strongest attentional modulation for each band, namely the superior occipital cortex for the alpha band and the middle occipital cortex for the gamma band (see Fig 2). Alpha and gamma asymmetry were not correlated with each other (r = -0.148, *p* = 0.47). We then correlated alpha and gamma asymmetry with the volumetric asymmetry of the three SLF branches.

**Fig 4:**
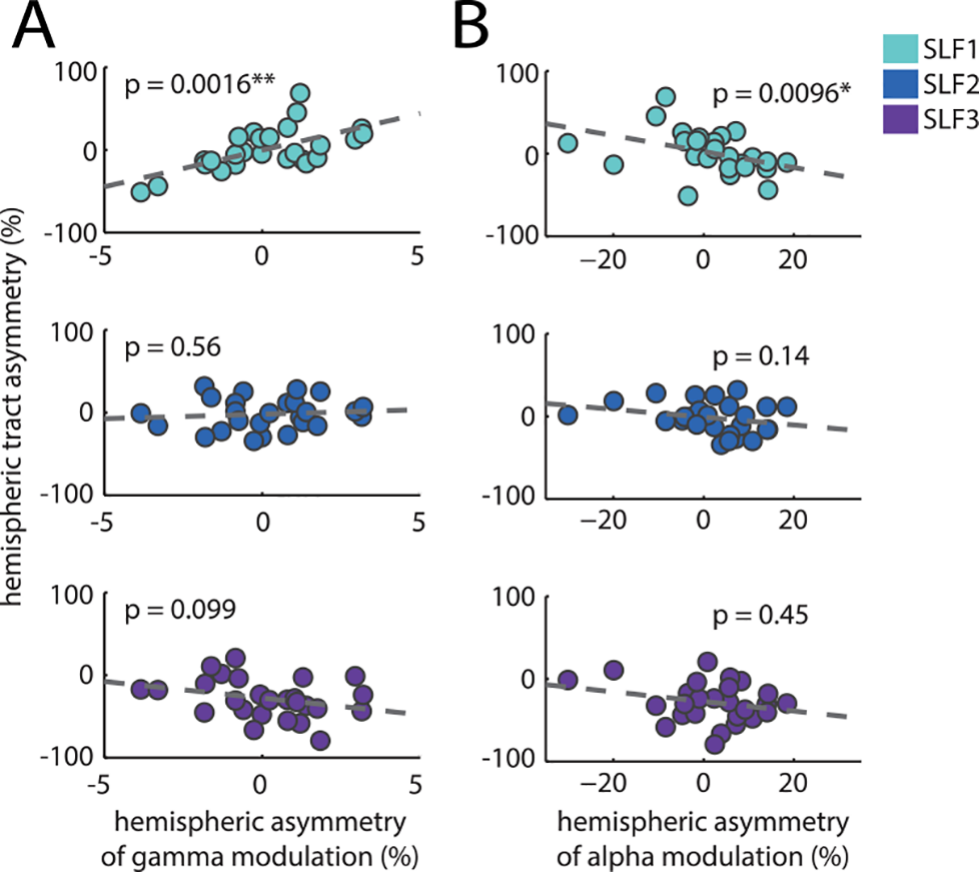
(A) Correlation of gamma modulation asymmetry in the middle occipital cortex (see Fig 2) with volumetric asymmetry of the three SLF branches. The gamma modulation asymmetry was calculated by comparing the degree of attentional modulation (left versus right spatial cue) in the right versus the left hemisphere. In the case of the SLF1, gamma modulation asymmetry was strongly positively correlated with volumetric hemispheric asymmetry (*p* = 0.0016, significant at the *p* < 0.005 level after Bonferroni correction for three comparisons). Neither SLF2 nor SLF3 showed such a correlation. (B) The same correlations but for alpha modulation asymmetry in superior occipital cortex (see Fig 2). Only SLF1 volumetric hemispheric asymmetry showed a significant negative correlation with alpha modulation asymmetry (*p* = 0.0096, significant at the *p* < 0.05 level after Bonferroni correction for three comparisons). As such, subjects with stronger left than right tracks in SLF1 were able to modulate the left compared to right hemisphere alpha and gamma power to a larger degree.

Our main finding (Fig 4A, top panel) shows that gamma modulation asymmetry was strongly positively correlated with SLF1 hemispheric asymmetry (r = 0.596, r^2^ = 0.36, *p* = 0.0016, Spearman, significant at the *p* < 0.005 level after Bonferroni correction for three comparisons). This demonstrates that subjects who displayed relatively greater gamma modulation in the left hemisphere than in the right hemisphere also had relatively greater tract volume in the left than in the right hemisphere (and vice versa). No correlation was observed with SLF2 or SLF3 (in all cases *p* > 0.05 without Bonferroni correction).

Our second main finding (Fig 4B, top panel) shows that alpha modulation asymmetry was strongly negatively correlated with SLF1 hemispheric asymmetry (r = -0.503, r^2^ = 0.25, *p* = 0.0096, Spearman, significant at the *p* < 0.05 level after Bonferroni correction for three comparisons). This means that subjects who displayed relatively greater alpha modulation in the left hemisphere than in the right hemisphere also had relatively greater tract volume in the left than in the right hemisphere. The difference in the signs of the correlation is explained by alpha power decreasing and gamma power increasing contralateral to attention (see Materials and Methods for detailed explanation). No correlation was observed with SLF2 or SLF3 (in all cases, *p* > 0.1 without Bonferroni correction). This is evidence that individual differences in SLF1 hemispheric asymmetry predict individual differences in the top-down modulation of neuronal synchronization in both the alpha and gamma band.

To determine whether target-driven reorienting produced an asymmetry in the gamma band, we computed a reorienting index (RI) analogous to the AMI, according to the formula *RI*_*j*_ *=* 100% * *(Power*_*Attention both target left*,*j*_ *—Power*_*Attention both target right*,*j*_ *) / (Power*_*Attention both target left*,*j*_ *+ Power*_*Attention both target right*,*j*_ *)*, for the gamma-band data in the post-stimulus window. This did not reveal a pattern of lateralized modulation, and no correlation was observed with any SLF branch (*p* > 0.1 without Bonferroni correction in all cases).

### Occipital Gamma Modulation Asymmetry Predicts Reaction Time Benefit from Spatial Cues

**Fig 5:**
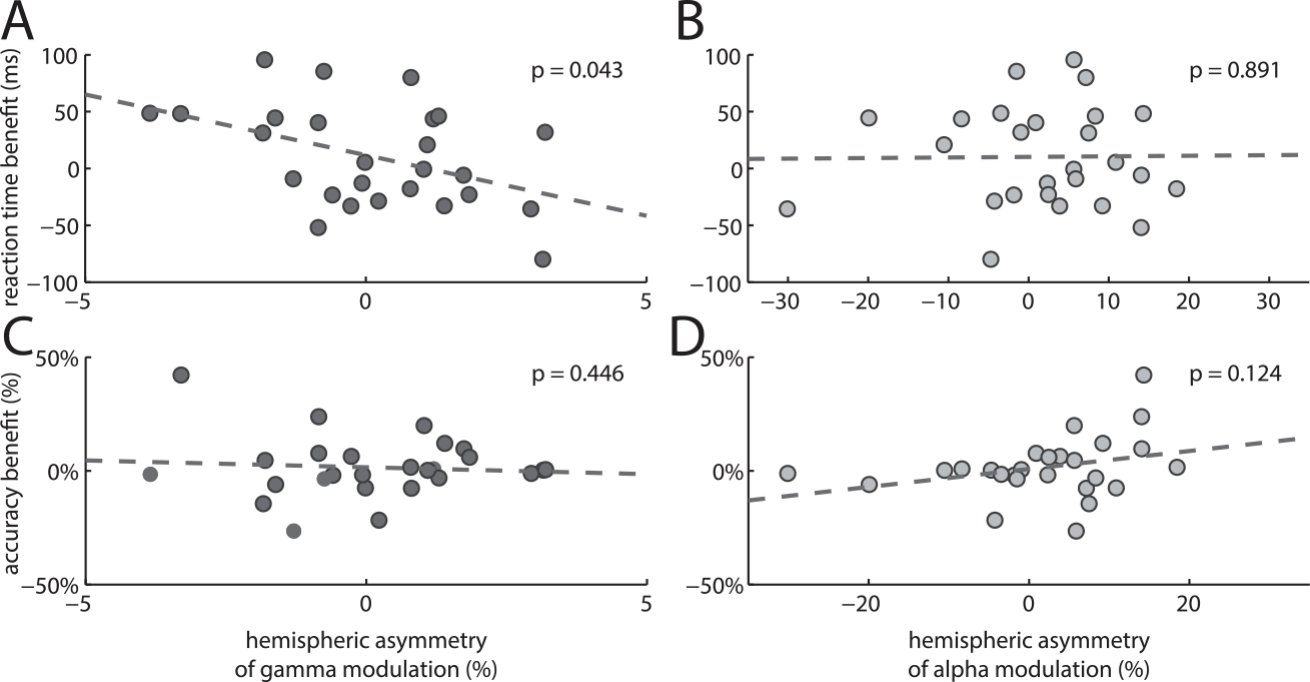
Correlation of oscillatory hemispheric asymmetry with behavioral measures. (A) Correlation of gamma asymmetry with reaction time benefit from left versus right spatial cues. Subjects benefitted relatively more from (i.e., responded faster to) a spatial cue contralateral to the hemisphere in which they showed greater gamma modulation. This supports the notion that gamma leads to enhanced stimulus processing. (B) As A, but for alpha asymmetry. Here no relationship was observed. (C) Accuracy benefit from left versus right spatial cues. No relationship was observed between accuracy benefit and gamma asymmetry. (D) As C, but for alpha asymmetry. Again, no relationship was observed.

Having demonstrated a link between hemispheric asymmetry of SLF1 and both anticipatory alpha and stimulus-induced gamma band modulations in visual cortex, we further tested if these effects were predictive of subjects’ task performance. Accordingly, we quantified the degree to which subjects benefitted (in terms of reaction time and accuracy) from a left versus a right cue in comparison to the control condition with no spatial cue (see Materials and Methods). This hemifield specific asymmetry of the cueing benefit correlated with the hemispheric asymmetry of occipital gamma power modulation (*ΔAMI*; Fig 5A, r = -0.40, *p* < 0.05) but did not correlate with alpha power modulation (Fig 5B, r = 0.03, *p* = 0.89). The negative correlation value means that subjects with relatively stronger gamma modulation in the left occipital cortex than in the right occipital cortex benefitted more from a right cue than a left cue. This is fully commensurate with the notion that visual cortical gamma modulation in the hemisphere contralateral to target presentation boosts effective synaptic gain and thus enhances stimulus processing. No correlation was observed between accuracy benefit and hemispheric asymmetry of occipital gamma modulation (Fig 5C, r = 0.16, *p* = 0.45) or alpha modulation (Fig 5D, r = 0.31 *p* = 0.12).

Participants also performed a behavioral “landmark” task outside the MEG, designed to test spatial perceptual and motor response biases in the absence of directed attention [29–31]. Performance on this task was found not to correlate with hemispheric asymmetry of any SLF branch (see S1 Text and S3 Fig).

### Gamma Modulation in the Superior Frontal Cortex Correlates with SLF1 Hemispheric Asymmetry

**Fig 6:**
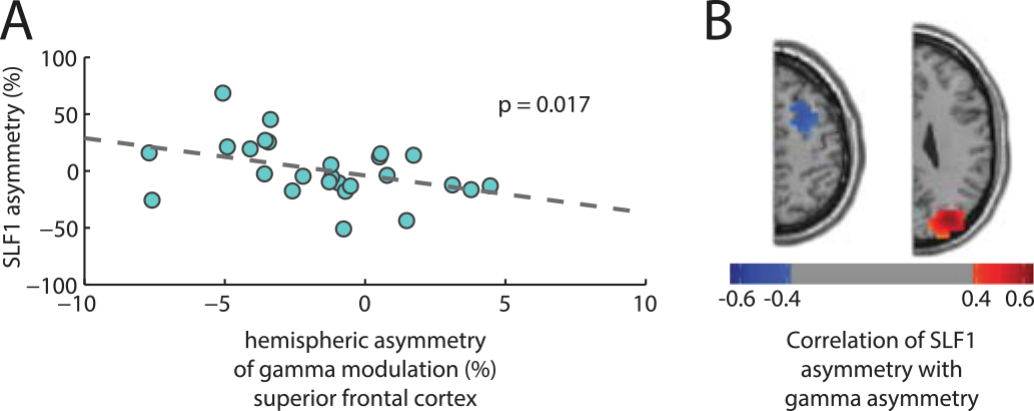
(A) Correlation of SLF1 asymmetry with gamma-band hemispheric asymmetry in superior frontal cortex (−26 +6 +56; as defined in [19]). A clear negative correlation is observed, which—notably—is opposite in sign to the correlation between SLF1 asymmetry and occipital gamma modulation asymmetry. (B) Topographic map of correlation of gamma-band hemispheric asymmetry with SLF1 asymmetry. Map is thresholded at p < 0.05, uncorrected. MNI coordinates for slices: +66, +54. A sign reversal is evident for the frontal grid points compared to the posterior grid points. Whereas stronger gamma modulation in the occipital cortex is associated with a relatively larger ipsilateral SLF1, in the frontal cortex it is associated with a relatively larger contralatera*l* SLF1.

Although evidence exists for behaviorally relevant modulation of alpha and gamma oscillations in the occipital cortex [13,14], there is evidence that the top-down control signals that produce these modulations originate in the frontal cortex [16,18]. Given that gamma oscillations likely represent a general-purpose mechanism for effective communication [32], we further investigated whether SLF1 asymmetry predicted hemispheric asymmetry of gamma oscillations in prefrontal regions. To do this, we predefined two frontal ROIs: first, the FEF as defined by a meta-analysis of saccade studies [33] and, second, an adjacent region in the superior frontal cortex that has been identified as part of a frontoparietal network underpinning spatial attention and working memory [19,20]. To our surprise, hemispheric gamma modulation asymmetry (delta AMI) was found to correlate strongly with SLF asymmetry in the latter ROI (Fig 6A, r = -0.47, *p* = 0.017). Notably, the correlations in superior frontal cortex are negative, while they are positive in the occipital cortex. Fig 6B shows statistical maps of the correlation of SLF1 asymmetry with gamma asymmetry for every grid point. Grid points in the frontal cortex show negative correlations, and grid points in the occipital cortex show positive correlations. This means that those subjects with a greater left than right SLF1 volume actually displayed relatively greater gamma modulation in the right than left superior frontal cortex.

For the FEF as defined from the saccade literature, no correlation was observed with respect to hemispheric gamma modulation asymmetry (r = 0.35, *p* = 0.08). Neither ROI showed a correlation with hemispheric alpha modulation asymmetry (r = -0.33, *p* = 0.097, and r = 0.02, *p* = 0.92, respectively). No correlations were observed between SLF2 or SLF3 asymmetry and hemispheric alpha or gamma modulation asymmetry in the above ROIs (*p* > 0.15 in all cases).

Finally, we computed functional connectivity values between the superior frontal and occipital ROIs within the left and right hemispheres for each subject using power envelope correlations [34] and correlated the hemispheric asymmetry in functional connectivity with asymmetry of the SLF branches. No correlation was observed for the alpha or the gamma band data (all *p* > 0.1).

## Discussion

As reported in numerous studies, we have shown that stimulus-induced gamma band activity increases with spatial attention. Further, alpha oscillations decrease in anticipation of an upcoming stimulus. Importantly, we have now demonstrated a relationship between hemispheric asymmetry of the medial branch of the SLF (SLF1) and individual differences in the ability to exert top-down control over both anticipatory-alpha and stimulus-induced gamma oscillations. To our knowledge, this is the first evidence demonstrating that individual differences in frontoparietal white matter tracts predict the ability to modulate occipital cortical oscillations. This is strong evidence that the SLF1 is a structural pathway mediating top-down signals that control attentional modulations in visual cortex by modulating neuronal synchronization.

There is evidence suggesting that attention-modulated neuronal synchronization in the gamma band increases effective synaptic gain, and this synaptic gain increase enhances the impact of a neuronal population on connected downstream regions [1,35].Crucially, the ability to modulate gamma band activity in the present study was found to be predicted by the SLF1. Top-down signals from frontal cortex may thus serve to enhance gamma band synchronization and thus effective communication between visual cortex and downstream brain regions [2]. Emphasizing the relevance of these connections, hemispheric gamma band asymmetry was itself found to predict reaction times on the behavioral cueing task. This implies a causal chain by which a structural feature—hemispheric SLF1 asymmetry—can impact behavioral outcomes via its effect on neuronal dynamics. In contrast, no relationship was found between alpha oscillations and accuracy, in contrast to previous reports [36,37]. Gamma power has previously been shown to lock to the phase of ongoing alpha oscillations [38], suggesting an intimate relationship between bottom-up drive (indexed by the former) and pulsed inhibition (indexed by the latter). The present findings suggest that attentional modulation of alpha and gamma oscillations may not be related in such a simple fashion. The direct relationship between alpha and gamma oscillations should be a topic for future studies.

The relationship between hemispheric asymmetry of tract volumes and modulation of occipital cortical oscillations warrants further investigation. We propose that larger tract volume results in a higher fidelity of the top-down signal. A larger number of top-down connections from frontal control regions could result in a stronger propagation of the top-down signal by increased signal transmission. Tract volume is likely to depend on several factors including number of axons, proportion of myelinated axons, and axonal diameter [21]. Future work should therefore focus on identifying contributions of these factors to the effect on oscillatory modulation observed in the present study.

A previous HARDI study from Thiebaut de Schotten and colleagues found that SLF2 asymmetry predicted attentional task performance, whereas in the present study we found a relationship with SLF1. This is most likely explained by differences in the tasks. Although both studies used Posner paradigms [39], Thiebaut de Schotten and colleagues used 50% cue validity (Thiebaut de Schotten et al. [21], Supplemental Materials, page 12). Accordingly their subjects may have adopted a more stimulus-driven strategy engaging the ventral attentional network [15], consistent with the notion that the SLF2 supports communication between the dorsal and ventral networks [21]. The present study uses 100% valid cueing allowing preallocation of attention and likely engaging the dorsal attentional network. The present findings complement and extend these previous findings, demonstrating that in the context of high cue validity the dorsal network (and thus SLF1) is more strongly implicated.

The present study demonstrated that frontal top-down signals propagated via SLF1 impact visual cortical oscillations. Data from nonhuman primates implicate beta-band (18–34 Hz) oscillations in the FEF as controlling shifts of covert attention [40], and entrainment of 30 Hz activity in FEF using TMS has been shown to enhance visual perceptual sensitivity on a visual detection task in humans [41]. However, and consistent with our main hypotheses, initial sensor-level analysis of the MEG data (Fig 2) rather revealed robust attentional modulation during the cue-target interval in the alpha band and during the post-stimulus period in the gamma band, consistent with previous studies [1,6–11]. As well as the beta band, there is also some evidence that gamma-band phase interregional synchronization between frontal and posterior cortex is modulated by direction of attention [6], making this another candidate mechanism for top-down control. Future studies should attempt to further elucidate the precise form these attentional top-down control signals take.

The sources of the modulation of anticipatory alpha and stimulus-induced gamma oscillations were identified in the occipital cortex. The degree to which this attentional modulation was stronger in one hemisphere correlated strongly with hemispheric asymmetry of SLF1 volume. Crucially, however, a region in the superior frontal cortex also showed a similar effect in the gamma band, but with the opposite sign. This means that—whereas greater SLF1 volume in the left hemisphere (versus right) predicted stronger attentional gamma modulation in the left occipital cortex (versus right)—in the superior frontal cortex, greater SLF1 volume predicted weaker ipsilateral gamma modulation as compared to contralateral. Since modulation asymmetry is a measure of interhemispheric difference in modulation, this suggests a coupling of attentional gamma modulation between frontal cortex and contralateral visual cortex. Some evidence of such contralateral connections has been seen in previous TMS studies [18,42]. Furthermore, besides being the hypothesized frontal terminus of SLF1 [21], this frontal region is also adjacent to the human frontal eye field [33], a key node in the dorsal attentional network known to be involved in top-down allocation of attention [43–45]. Notably, one TMS study explicitly demonstrated a link between the disruptive effect of TMS to the right FEF on a visual perception task and properties of the SLF1 [23], suggesting that this white-matter tract indeed serves as the structural basis for communicating signals from FEF to other nodes in the dorsal attentional network.

Whilst we demonstrate a role for a cortico-cortical connection in top-down control of attentional oscillations, it is important to also consider cortico-subcortical connections. A recent nonhuman primate study demonstrated functional and structural connectivity between pulvinar and several visual areas, with the former serving to synchronize neocortical regions during a visuospatial attention task [46]. The cortico-cortical pathway we report on should be considered complementary to the subcortical pathway. The pulvinar may drive local synchrony between visual cortical regions preferentially during attention, whilst the frontal cortex provides top-down control signals that boost or attenuate the amplitude of attentionally relevant oscillations in response to task demands. Delineation of the respective contributions of both cortico-cortical and cortico-subcortical pathways should be the object of further study.

In conclusion, our data demonstrate for the first time (as far as we are aware) evidence for a cortico-cortical pathway providing top-down control of attentional modulations of behaviorally relevant neuronal oscillations in occipital cortex. This provides experimental support for the notion that modulation of visual cortical oscillations—and thus of effective synaptic gain—is the mechanism by which the dorsal attentional network asserts goal-directed attention.

## Materials and Methods

### Subjects

Twenty-eight right-handed subjects (15 males, 13 females, mean age of 24 y and 5 mo) participated in the experiment. All subjects underwent standard screening procedures for MEG and MRI. All experiments were carried out in accordance with the Declaration of Helsinki and following ethical approval by the local ethics board (CMO region Arnhem-Nijmegen, CMO2001/095). One subject elected not to complete the diffusion scanning, meaning diffusion data were unavailable, and for one subject SLF branches could not be reconstructed. Therefore, the analyses were conducted on the remaining 26 datasets.

### MEG

#### Behavioral cueing task

Subjects performed a cued visuospatial attention task (Fig 1A). At all times, two luminance pedestals were present on the screen, 3.2° of visual angle below the horizontal meridian and 4.8°of visual angle to the left and right of the vertical meridian. Each trial began with the presentation of visual cues presented to the left and right of a central fixation dot. One patch served as a target and the other as a distractor. These cues instructed the subject to either attend to the left pedestal, the right pedestal, to attend to both pedestals, or to passively view without responding. Following a 1,500 ms delay interval, a pair of target Gabor patches with a spatial frequency of four cycles per degree visual angle were presented at each luminance pedestal for 60 ms, followed by a 60 ms mask. The target patch was tilted either 45° clockwise or anticlockwise. The distractor patch was either horizontal or vertical. Following “attend left” and “attend right” cues, the target patch was always presented at the cued luminance pedestal (i.e., 100% valid cues). In the “attend both” and “passive viewing” conditions, the target appeared on the left or right with equal probability. Subjects were instructed to report the orientation of the target patch by a button press with the right hand (index finger = clockwise, middle finger = anticlockwise), except in the passive viewing condition in which no button press was required. No instruction was given regarding prioritization of speed over accuracy. Subjects completed 13 blocks of 40 trials and took short breaks between blocks. The total task duration was approximately 50 min.

#### MEG data acquisition

Continuous whole-brain activity was recorded using a CTF 275-channel MEG system (CTF MEG systems, VSM MedTech) at a sampling rate of 1200Hz. Ear canal and nasion markers were used to continuously monitor head position via a real-time head localizer [47]. When head position deviated >5 mm from the origin position (marked at the commencement of recording), subjects readjusted to the origin position at the next block break. An EyeLink 1000 eyetracker (SR Research, Ottawa, Canada) was used to continuously track the left eye to detect eye blinks and saccades.

#### MEG data analysis

MEG analysis was performed using the Fieldtrip Matlab toolbox [48]. We first applied automatic artifact rejection to remove trials containing SQUID jumps and muscle activity; in the former case, we applied a nine-sample median filter to the data and then z-transformed and thresholded the data to detect high-amplitude changes in MEG channels; in the latter case, we bandpass-filtered the data between 110 and 140 Hz (frequencies in which electromyographic activity is visible) using an eighth-order butterworth filter and then z-transformed and thresholded the data. In both cases, trials containing high z-values were marked and discarded from subsequent analysis. We then manually inspected the remaining trials for blinks and horizontal eye movements (using the eyetracker data only), and trials containing either were also discarded. In all manual artifact rejection steps, experimenters were blind as to the experimental condition. A total of 20 ± 11% of trials (mean ± standard deviation) were discarded because of artifacts.

First sensor-level MEG analysis was carried out to identify attentional modulation of power. ICA was performed on the sensor-level data to remove heartbeat-related activity prior to performing time-frequency analysis. For low frequencies (2–35 Hz), time-frequency analysis using an FFT approach was performed using a 500 ms sliding time window multiplied with a Hanning taper. This was moved across the data in 50 ms steps. For high frequencies (30–100 Hz), a set of seven orthogonal Slepian tapers (resulting in 15 Hz spectral smoothing) were applied to data segments of a 250 ms sliding time window prior to the FFT. AMIs were computed for each sensor *j* and frequency *k* according to the formula below:

$$AMI_{j}=100\%\;*\;\left(Power_{Attention\;left,j,k}-Power_{Attention\;right,j,k}\right)\;/\;\left(Power_{Attention\;left,j,k}\;+\;Power_{Attention\;right,j,k}\right)$$

All subsequent MEG analyses to test the main hypotheses were performed at the source level using dynamic imaging of coherent sources (DICS, [26]). No ICA was performed on these data prior to beamforming. A single-shell head model [49] was constructed from the anatomical MRI. A template grid with 6 mm^3^ spacing was constructed using an MNI template brain. This grid was symmetrical with respect to the sagittal axis of the MNI brain. From this, single subject grids were produced by warping the individual anatomical scans to this template and applying the inverse warp to the template grid. This produced source-level data aligned across subjects in MNI space.

The source analyses for the alpha- and gamma-band data were conducted separately. For the alpha band, 1,000 ms data segments from the pre-cue period (−1,000–0 ms pre-cue) and cue-target interval (350–1,350 ms post-cue) were used. These time windows were preselected based on [18], in which a similar task was used. Cross-spectral densities were computed using a set of three orthogonal Slepian tapers with a 10 Hz center frequency to produce 2 Hz frequency smoothing [50] (i.e., 8–12 Hz band). For the gamma band, 400 ms data segments from the cue-target interval (900–1,300 ms post-cue) and post-stimulus period (1,700–2,100 ms post-cue) were used. A set of 15 orthogonal Slepian tapers with a 70 Hz center frequency produced 20 Hz frequency smoothing (i.e., a 50–90 Hz band). All time windows were preselected based on [18], in which a similar task was used. For both the alpha and gamma frequency bands, a common spatial filter was constructed using data from all time windows and all trial conditions, from which an AMI was computed for each grid point (*j*) according to the formula:

$$AMI_{j}=100\%\;*\;\left(Power_{Attention\;left,j}-Power_{Attention\;right,j}\right)\;/\;\left(Power_{Attention\;left,j}\;+\;Power_{Attention\;right,j}\right)$$

These AMI maps were interpolated to a template anatomical brain.

To quantify the reliability of the sources reflecting the modulations of alpha and gamma power, we used a cluster-based permutation approach, which effectively controls for multiple comparisons over grid points [27]. Using this approach, we carried out a paired-sample *t*-test on the averaged preselected frequency bands and time windows (see above), comparing mean power values from “attend left” and “attend right” trials. T-scores exceeding a given threshold (*p* < 0.05, uncorrected) were clustered on the basis of spatial adjacency, and the summed t-value from the cluster was computed. The data labels were then randomized 10,000 times, and a cluster t-value was computed for each randomization, creating a reference distribution of cluster t-values under the null hypothesis of no difference between the “attend left” and “attend right” conditions. The initial cluster t-value was then evaluated with respect to this reference distribution.

To correlate individual differences in the topography of modulations of alpha and gamma power with SLF properties, a modulation asymmetry score was calculated for each subject. This score expresses in a single number, for a given subject and anatomical ROI, whether that subject demonstrates a greater attentional power modulation in the left or right hemisphere. Since both alpha and gamma modulation demonstrate hemispheric specificity and thus modulate in opposite directions in each hemisphere, it is necessary to reverse the sign of the modulation in one hemisphere to compare the two. Accordingly, we computed modulation asymmetry as follows:

$$\Delta AMI=\left(-AMI_{left,j}\right)-AMI_{right,j}$$

In order to test our main hypothesis, MA scores were created by averaging the AMI values across ROIs defined using the AAL atlas, a standard atlas used for anatomical labeling based on parcellation of a high-resolution MNI image [51]. The AAL atlas demarcates 45 regions per hemisphere based on the main cortical sulci. For alpha oscillations, since the peak AMI values were found in left and right superior occipital cortex, we considered the average AMI values across all grid points within these regions and computed the MA according to the above formula. Similarly, since the strongest gamma AMI values were found in the left and right middle occipital cortex, we considered the average AMI across all grid points within these two regions and computed the MA according to the same formula. This approach was taken in order to create anatomically mirror symmetric regions from which to calculate functional asymmetry. Additionally, two frontal ROIs were defined based on MNI coordinates reported in previous literature; firstly, we took the coordinate of the “classical” FEF identified from a meta-analysis of saccade studies [33] (left hemisphere: −32 −2 46; right hemisphere: 32 −2 46); secondly, we took the coordinate of an adjacent region in the superior frontal cortex known to be connected to frontoparietal white matter and to emerge in parallel with it during development [19] (left hemisphere: −26 6 56; right hemisphere: 26 6 56).

To illustrate the reversal of sign between correlations of frontal and gamma modulation asymmetry and SLF1 asymmetry, modulation asymmetry was calculated separately for every pair of grid points (mirror symmetric about the sagittal midline) and thresholded at *p* < 0.05, uncorrected (Fig 6B). This illustrative analysis is intended only to describe the two observed effects that were validated using a ROI-based approach.

#### Behavioral data analysis

To quantify the degree to which attentional cues resulted in behavioral benefits (i.e., speeded responses), we made use of behavioral data from “attend left,” “attend right,” and “attend both” trials. We quantified the “cueing benefit” of a left cue according to the formula:

$$cueing\;benefit_{left}=RT_{attend\;left,\;target\;left}-RT_{attend\;both,\;target\;left}$$

Similarly, for a right cue, we used the following:

$$cueing\;benefit_{right}=RT_{attend\;right,\;target\;right}-RT_{attend\;both,\;target\;right}$$

We then calculated the degree to which this cueing benefit was biased in favor of one hemifield by computing:

$$cueing\;benefit\;asymmetry=cueing\;benefit_{left}-cueing\;benefit_{right}$$

#### Connectivity analysis

To investigate the relationship between within-hemisphere structural and functional connectivity, we computed power envelope correlations using Hipp’s single-trial orthogonalization method [34]. We used the same regions in superior frontal cortex (left and right hemisphere) shown above to relate to hemispheric asymmetry of alpha and gamma and reported previously in [19]. From these, we computed connectivity with the superior frontal cortex (for the alpha band) and middle occipital cortex (gamma band), as these regions had demonstrated the strongest attentional power modulations (Fig 2) within a hemisphere (i.e., left frontal to left occipital regions and right frontal to right occipital regions). As previously, we averaged across the entire region as delineated by the AAL atlas [51]. We then computed connectivity asymmetry by calculating the difference in connectivity scores between the left and right hemispheres for each subject and correlated those difference scores with the volumetric asymmetries of the SLF branches.

### Diffusion MRI

#### Acquisition

Diffusion data were acquired using a 3T Siemens Skyra system (Siemens, Erlangen, Germany). Sequence parameters were as follows: anterior-posterior phase encoding, voxel size 2.2 * 2.2 * 2.2 mm, matrix size 100 * 100, slices 64, NEX 1, TR = 10,500 ms, TE = 90.0 ms, b-value 1,500 s / mm^2^, 60 diffusion-weighted directions, and eight non-diffusion-weighted volumes. The first non-diffusion-weighted volume served as an anatomical reference for eddy current correction. A high-resolution T1-weighted image (TR = 2,300 ms, TE = 3.03 ms, flip angle = 8°, 192 sagittal slices, in plane voxel size = 1 × 1 × 1 mm, FoV = 256 × 256 mm) was also acquired for MEG source analysis.

#### Preprocessing

Diffusion MRI analysis procedure closely followed that of Thiebaut de Schotten and colleagues [21]. Eddy current correction was performed in FSL [52]. White-matter orientation estimation and diffusion tractography were performed using StarTrack (http://www.natbrainlab.com). Spherical deconvolution [24,25] with modified Richardson-Lucy damping [28] was used for optimal estimation of fiber orientations in voxels containing crossing fiber populations. Both absolute and relative thresholds were used to exclude spurious maxima of fiber orientation distributions.

#### Tractography and SLF dissection

Whole-brain tractography was performed starting from every voxel with at least one fiber orientation as a seed voxel. From these voxels and for each fiber orientation, a modified fiber assignment using a continuous tracking algorithm was used to reconstruct streamlines by sequentially piecing together discrete and shortly spaced estimates of fiber orientation forming continuous trajectories. When entering a region with crossing white-matter bundles, the algorithm followed the orientation vector of least curvature. Streamlines were halted when a voxel without fiber orientation was reached or when the curvature between two steps exceeded a threshold of 45°.

A previously validated method [21] was used to dissect the three branches of the SLF (see Fig 3A). Streamlines were designated as “SLF” if they (A) passed through parietal cortex parallel to the posterior commissure in the coronal plane and (B) passed, respectively, through the superior frontal gyrus (SLF1), the middle frontal gyrus (SLF2), or the precentral gyrus (SLF3) parallel to the anterior commissure in the coronal plane. Fibers extending to the temporal lobe or to the internal or external capsules were excluded.

For each SLF branch in each hemisphere, a binary “visitation map” was created: every voxel was assigned a value of 1 if streamlines from the SLF branch passed through that voxel; otherwise, a 0 was assigned. These binary maps were then normalized to MNI space and smoothed with smoothing kernel of 4 mm^3^ FWHM using SPM (http://www.fil.ion.ucl.ac.uk/spm). For SLF1, SLF2, and SLF3, a hemispheric asymmetry index was computed analogously to the AMI, namely as follows:

$$SLF\;asymmetry=100\%\;\left(Volume_{Left\;branch}-Volume_{Right\;branch}\right)\;/\;\left(Volume_{Left\;branch}+Volume_{Right\;branch}\right)$$

Here, *volume* refers to the number of voxels intersected by that branch.

#### Data availability

The dataset used to reach the conclusions drawn in this study is deposited in the Dryad Data Repository: https://datadryad.org/resource/doi:10.5061/dryad.bt7v0 [53].

## Supporting Information

S1 Fig Dependent samples t-statistics contrasting alpha power during cue-target interval on attention left trials and attention right trials. Values are masked with a cluster-based permutation test *p* < 0.025 (two-tailed), thus controlling for multiple comparisons. Images are interpolated onto an MNI template brain.
(EPS)

S2 Fig Dependent samples t-statistics contrasting gamma power during post-stimulus interval on attention left trials and attention right trials. Values are masked with cluster-based permutation test *p* < 0.025 (two-tailed), thus controlling for multiple comparisons. Images are interpolated onto an MNI template brain.
(EPS)

S3 Fig Landmark task paradigm. Subjects were instructed to report either the longer or shorter side of a briefly presented vertically bisected line.
(EPS)

S1 Text Description of landmark task and results. (DOCX)

## Abbreviations

AMI: attentional modulation index
DICS: dynamic imaging of coherent sources
EEG: electroencephalography
FEF: frontal eye field
HARDI: high angular resolution diffusion imaging
IPS: intraparietal sulcus
MEG: magnetoencephalography
MNI: Montreal Neurological Institute
MR: magnetic resonance
RI: reorienting index
ROI: region of interest
SLF: superior longitudinal fasciculus
TMS: transcranial magnetic stimulation
