# Supplementary material for: Frontoparietal Structural Connectivity Mediates the Top-Down Control of Neuronal Synchronization Associated with Selective Attention
Source: PLoS Biol. 2015 Oct 6;13(10):e1002272. doi: 10.1371/journal.pbio.1002272 (PMC4595220; doi:10.1371/journal.pbio.1002272)
Supplement: S1 Text — (DOCX) [file pbio.1002272.s004.docx]

**Landmark Task**

In addition to assessing top-down control of alpha and gamma oscillations and benefit of spatial cues using a cued attention task in the MEG, we also assessed spatial biases using a modified line bisection task known as the landmark task [1,2] (see Figure S3). Full details of this task can be found in [3]. Briefly, subjects were shown asymmetric horizontal lines intersecting a central fixation cross, and were instructed to report the side of either the longer or the shorter line segment (the instruction changed on every block). An adaptive staircase procedure was used to determine both the perceptual bias (PB), the mean deviation of subjective equality from true equality, and response bias (RB), the mean probability of responding opposite to the perceived midpoint. PB and RB are mathematically independent [2]. This task was repeated 8 times, with 42 trials per repetition. Performance on this task was found not to correlate with hemispheric asymmetry of any SLF branch (Perceptual bias: SLF1; r = 0.084, p = 0.68, SLF2; r = 0.024, p = 0.91, SLF3; r = -0.23, p = 0.26. Response bias: SLF1; r = 0.24, p = 0.23, SLF2; r = -0.27, p = 0.18, SLF3; r = -0.30, p = 0.14).

**Supporting References**

1. Milner AD, Brechmann M, Pagliarini L. To halve and to halve not: An analysis of line bisection judgements in normal subjects. Neuropsychologia. 1992;30: 515–526.

2. Bisiach E, Ricci R, Lualdi M, Colombo M. Perceptual and response bias in unilateral neglect: two modified versions of the Milner landmark task. Brain Cogn. 1998;386: 369–386.

3. Szczepanski SM, Kastner S. Shifting attentional priorities: control of spatial attention through hemispheric competition. J Neurosci. 2013;33: 5411–21. doi:10.1523/JNEUROSCI.4089-12.2013
